# Effects of VRK2 (rs2312147) on White Matter Connectivity in Patients with Schizophrenia

**DOI:** 10.1371/journal.pone.0103519

**Published:** 2014-07-31

**Authors:** Hoyoung Sohn, Borah Kim, Keun Hyang Kim, Min-Kyoung Kim, Tai Kiu Choi, Sang-Hyuk Lee

**Affiliations:** Department of Psychiatry, CHA Bundang Medical Center, CHA University, Seongnam-si, Gyeonggi-do, Republic of Korea; University of California, San Francisco, United States of America

## Abstract

Recent genome-wide association studies of schizophrenia reported a novel risk variant, rs2312147 at vaccinia-related kinase 2 gene (VRK2), in multiple Asian and European samples. However, its effect on the brain structure in schizophrenia is little known. We analyzed the brain structure of 36 schizophrenia patients and 18 healthy subjects with regard to rs2312147 genotype groups. Brain magnetic resonance scans for gray matter (GM) and white matter (WM) analysis, and genotype analysis for VRK2 rs2312147, were conducted. The Positive and Negative Syndrome Scale and the Digit Symbol Test were assessed for schizophrenia patients. There was no significant difference in either GM volume or WM connectivity with regard to rs2312147 genotype in healthy subjects. In contrast, we found significant differences in the WM connectivity between rs2312147 CC and CT/TT genotype groups of schizophrenia patients. The related brain areas included the splenium of corpus callosum, the left occipital lobe WM, the internal capsule (left anterior limb and right retrolenticular part), the bilateral temporal lobe WM, the left fornix/stria terminalis, the left cingulate gyrus WM, and the left parietal lobe WM. Voxelwise correlation analysis revealed that the Digit Symbol Test scores (age corrected) correlated with the fractional anisotropy in WM tracts that previously showed significant group differences between the CT/TT and CC genotypes in the rs2312147 CT/TT genotype group, while no significant correlation was found in the CC genotype group. Our data may provide evidence for the effect of VRK2 on WM connectivity in patients with schizophrenia.

## Introduction

Recent developments in genome sequencing have made schizophrenia a hot topic in the genetics of neuropsychiatry through genome-wide association studies (GWASs). In recent years, GWASs and meta-analyses reported a group of common genetic variants significantly conferring the risk of schizophrenia in Europeans [1], [2], [3], [4], [5], [6], [7] and in Chinese [8], [9]. Among them, rs2312147 [C], located ∼50 kb upstream of vaccinia-related kinase 2 (VRK2), was identified as a novel risk of single nucleotide polymorphism (SNP) associated with schizophrenia in Europeans [2], [5], and Asians [9].

The VRK2 gene encodes a member of the vaccinia-related kinase family of serine/threonine protein kinases. Although the function of VRK2 in schizophrenia is not well known, VRK2 rs2312147 C allele was reported to show a significant association with schizophrenia [European combined samples: odds ratio (OR) = 1.09, P = 1.9×10^−9^ [5]; Asian combined samples: OR = 1.16, P = 4.24×10^−4^ [9]]and VRK2 gene expression was up-regulated in the blood cells of patients with schizophrenia [9]. Also, the tendency of the increments of VRK2 expression in the brains of schizophrenia patients can be found on the Stanley Medical Research Institute Online Genomics Database (http://www.stanleygenomics.org), though it was not statistically significant. In addition, several other studies have reported the possibilities of the relation between VRK2 and neurodevelopmental abnormalities, e.g., cortical dysplasia [11],[12],[13] and epilepsies [14]. The role of VRK2 in determining the magnitude of the stress response induced by hypoxia [15] or modulation of interleukin-1β transcriptional response [16] might implicate a probable connection to the pathophysiology of schizophrenia [17], [18], [19].

Genetic variants have been suggested to be associated with cognitive dysfunction in schizophrenia [20]. Of the many cognitive tests, the Digit Symbol Test (DST) [21] can differentiate healthy controls from people with schizophrenia and their close relatives better than other measures [22] and indexes poor prognosis and functional disability of patients with schizophrenia [23]. The processing speed, as measured by DST, may be considered a core cognitive deficit in schizophrenia and might mediate a broader diversity of cognitive disturbances [24], [25]. The DST engages a broad range of cognitive processes, making it suitable for identifying the fiber systems recruited by cognitive processes and their role on the speed and efficiency with performing the test [26]. It has been reported that DST scores were positively correlated with fractional anisotropy (FA) values of WM tracts in healthy young adults [26], patients with panic disorder [27], and patients with multiple sclerosis [28]. These findings indicated a potential relationship between cognitive processing speed, as assessed by the DST, and the structural integrity of WM tracts.

Even though Li et al. [9] reported the association between rs2312147 and brain structure in healthy subjects, little is known about the influence of rs2312147 on brain structural characteristics of patients with schizophrenia. Therefore, we conducted the present study to explore the association between rs2312147 and the brain structure of schizophrenia. We hypothesized that (1) WM integrity would differ according the genotype of VRK2 rs2312147 in patients with schizophrenia, and (2) WM integrity would be associated with cognitive function, as assessed by DST performance, in relation to the rs2312147 genotype. Additionally, we investigated the diagnosis-by-genotype interaction between schizophrenia and healthy subjects and the relationship between WM integrity and clinical severity. To exclude the possible effects of gray matter (GM) volume changes in corresponding brain regions, a GM volume analysis was conducted.

## Materials and Methods

### Ethics Statement

The CHA Bundang Medical Center Ethics Committee approved this study. All study procedures complied with the CHA Bundang Medical Center Institutional Review Board regulations, Declaration of Helsinki, and principles of Good Clinical Practice. Subjects did not receive payment for participation in the study. All participants had the cognitive capacity to understand the research protocol and gave their oral and written consent after receiving a full description of the study.

### Subjects and Clinical Assessment

Patients with schizophrenia were recruited by advertising at the outpatient clinics of authors (BK, TKC, and SHL), and HC subjects were recruited by public advertisement between January 2011 and December 2012. Patients with schizophrenia met the Diagnostic and Statistical Manual of Mental Disorders, 4th Edition, Text Revision (DSM-IV-TR) [29] criteria for schizophrenia, as diagnosed by experienced psychiatrists using the structured clinical interview to assess DSM-IV-TR (SCID) Axis I disorders [30]. Exclusion criteria for all subjects included any history of mood disorders, alcohol and substance abuse or dependence, mental retardation, current or past serious medical or neurological disorders, and contraindications to brain magnetic resonance imaging (MRI), including metal implants, and pregnancy.

Thirty-six schizophrenia patients and 18 healthy control (HC) subjects were investigated in this study. All subjects were 18 to 65 years old, of Korean descent, and right-handed. The overall study processes are detailed in Figure S1.

At the time of brain MRI scan, all patients were assessed for the clinical severity of positive and negative symptoms using the Positive and Negative Syndrome Scale (PANSS) [31] and cognitive function using the Korean version of the DST [21], the subset of the Korean-Wechsler Adult Intelligence Scale [32]. We used age-corrected scores for DST to exclude the effect of age-related slowing [33].

### Genotyping

Genomic DNA was extracted from blood (stored frozen) using a G-DEX II Genomic DNA Extraction Kit (Intron, Korea) according to the manufacturer’s protocol. SNP detection was based upon analysis of primer extension products generated from previously amplified genomic DNA using a chip-based MALDI-TOF mass spectrometry platform (Sequenom, Inc., CA). General procedures were performed according to the manufacturer’s standard protocol. The PCR reaction was performed in a volume of 5 µL containing 1× PCR buffer (Solgent, Korea), 25 mM MgCl_2_, 25 mM of each dNTP, 5 U/µl hTaq Polymerase (Solgent, Korea), 1 µM of each primer, and 5–10 ng/µl of genomic DNA. Primers were designed using Assay Designer 3.1 (Sequenom, Inc., CA) as rs2312147-F (5′-ACGTTGGATGTCCAGGCAGTAGTACCAAAG) and rs2312147-R (5′-ACGTTGGATGCTTTTTCATCAAGAACATTC). The reaction consisted of denaturation at 95°C for 15 min, followed by 45 cycles at 95°C for 20 s, 56° for 30 s, and 72°C for 1 min, with a final extension at 72°C for 3 min. Following PCR, unincorporated dNTPs were removed by adding 1.7 U/µl of shrimp alkaline phosphatase (Sequenom, Inc., CA) and incubating for 40 min at 37°C, followed by 10 min at 85°C for enzyme inactivation. In the homogeneous MassEXTEND reaction, the total volume of each reaction was 9 µL, including thermo sequenase (Sequenom, Inc., CA), an appropriate termination mix, and 20 µM of extension primer. The primer extension protocol was started at 94°C for 2 min, followed by 40 cycles at 94°C for 10 s, 52°C for 10 s, and 72°C for 10 s. After desalting the reaction product with SpectroCLEAN (Sequenom, Inc., CA), samples were dispensed on a 384-well SpectroCHIP array (Sequenom, Inc., CA) using SpectroJET (Sequenom, Inc., CA). SpectroCHIPs were analyzed with a MALDI-TOF MassARRAY system (Sequenom, Inc., CA) in the fully automated mode. After overall automatic measurements, any assays with bad peaks were checked again manually.

The allele frequencies of VRK2 rs2312147 for Asian populations have been reported as follows (http://www.hapmap.org): Japanese in Tokyo (JPT), C allele 72%, T allele 28%; Han Chinese in Beijing (CHB), C allele 71%, T allele 29%. It was expected that the TT genotype group would be much smaller than the CC or CT genotype groups among Korean subjects in this study. To increase the statistical power, for imaging analyses we divided our subjects into two groups of genotypes according to allele frequencies. As a result of the extremely low frequency of the TT genotype in our sample, to increase the statistical power, we compared imaging data between subjects who were homozygous for the C allele and subjects who were not.

### MRI Acquisition

All scans were performed on the same 3 Tesla GE Signa HDxt scanner (GE Healthcare, Milwaukee, WI, USA) equipped with an eight-channel phase array head coil at CHA Bundang Medical Center, CHA University. Parameters for three-dimensional T1-weighted fast spoiled gradient recalled echo (3D T1-FSPGR) images were as follows: repetition time (TR) = 16 ms, echo time (TE) = 4.3 ms, flip angle = 10°, field of view (FOV) = 25.6 cm, matrix = 256×256, slice thickness = 1.7 mm, and isotropic voxel size = 1×1×1 mm^3^. Diffusion-weighted images were acquired using an echo planar imaging sequence with the following parameters: TR = 17000 ms, TE = 108 ms, FOV = 24 cm, matrix = 144×144, slice thickness = 1.7 mm, and voxel size = 1.67×1.67×1.7 mm^3^. A double-echo option was used to reduce eddy-current-related distortions. To reduce the impact of spatial distortions, an 8-channel coil and array spatial sensitivity encoding technique (ASSET; GE Healthcare) with a sensitivity encoding (SENSE) speed-up factor of 2 was used. Seventy axial slices parallel to the anterior commissure–posterior commissure line covering the whole brain were acquired in 51 directions with b = 900 s/mm^2^. Eight baseline scans with b = 0 s/mm^2^ were also acquired. DT values were estimated from the diffusion-weighted images by using the least-squares method (approximate scan time = 17 min).

### Image Processing

The image processing for gray matter (GM) was performed on Statistical Parametric Mapping (SPM) 5 software (Wellcome Trust Centre for Neuroimaging, UCL, London, UK; http://www.fil.ion.ucl.ac.uk/spm) using the Voxel Based Morphometry (VBM) 5 toolbox (http://vbm.neuro.uni-jena.de/vbm), run on MATLAB 7.9 (MathWorks, Natick, MA, USA). The two-dimensional DICOM files of each brain were organized into volumetric three-dimensional files as NIFTI-1 (http://nifti.nimh.nih.gov) format using the MRIcron software package (http://www.sph.sc.edu/comd/rorden/mricron). During VBM preprocessing, converted T1 image files were segmented into GM, WM, and cerebrospinal fluid (CSF) compartments and normalized using a unified model [34]. Then, voxel values were modulated by Jacobian determinants derived from the spatial normalization, which allowed brain structures that had their volumes decreased after spatial normalization to have their total counts decreased by an amount proportional to the degree of volume discounted. The final voxel resolution after normalization was 1 mm^3^. Finally, the modulated GM partitions were smoothed with a 12-mm full width at half maximum Gaussian kernel, and used for statistical analysis. Additionally, global GM, WM and CSF volumes, as well as total intracranial volumes, were computed using the native-space tissue maps of each subject.

For WM, image processing of the FA data was carried out using Tract-Based Spatial Statistics (TBSS) version 1.2 implemented in FMRIB Software Library (FSL) [35] version 5.0 according to standard procedures [36]. First, DTI preprocessing, including skull stripping using the Brain Extraction Tool (BET) and Eddy current correction, were performed using the FSL. FA images were created by fitting a tensor model to the raw diffusion data. All subjects’ FA data were then aligned into the standard space (Montreal Neurologic Institute 152 standard) using the FMRIB’s Nonlinear Image Registration Tool (FNIRT). All transformed FA images were combined and applied to the original FA map, resulting in a standard-space version FA map. All transformed FA images were averaged to create a mean FA image, which was then thinned (skeletonized) to create a mean FA skeleton, taking only the centers of WM tracts. The skeleton was thresholded by FA>0.2 (TBSS default) to include only major fiber bundles.

### Statistical Analysis of the GM volume data

Using VBM5, independent t-tests were performed for comparisons of GM volumes between the rs2312147 CC and CT/TT genotype schizophrenia patients, as well as between the schizophrenia and HC subjects. An analysis of covariance (ANCOVA) was conducted to confirm whether the same focal GM volume differences would be found with covariates of no interest, such as age, sex, and intracranial volume (ICV). To investigate the diagnosis-by-genotype interaction between schizophrenia and HC groups, a two-way analysis of variance (ANOVA) with age, sex, and ICV as covariates was conducted. Within each genotype group for patients with schizophrenia, voxelwise correlations between regional GM volumes were evaluated, in addition to PANSS and DST scores. The statistical threshold was set at p<0.05, with family-wise error (FWE) correction for multiple comparisons at cluster level.

### Statistical Analysis of the DTI data

We performed a voxel by voxel statistical analysis to detect regions of significant differences in FA among the two diagnostic groups (schizophrenia vs. HC) and genotype groups (rs2312147 CC vs. CT/TT), respectively, using nonparametric permutation tests with a correction for multiple comparisons with FSL Randomise program [37]. We used permutation-based nonparametric inference within the framework of the general linear model tested with 10,000 permutations, including full correction for multiple comparisons over space, to achieve accurate inference and the significance level was set at *p*<0.05. We corrected multiple comparisons with threshold-free cluster enhancement (TFCE) [38], which enabled us to avoid making a random choice of the cluster-forming threshold, while preserving the sensitivity benefits of cluster-wise correction. In addition, ANCOVA, with age and sex as covariates, was conducted to confirm the effect of other variables on the results. To investigate the diagnosis-by-genotype interaction between schizophrenia and HC subjects, a two-way ANOVA with age and sex as covariates was conducted. We conducted correlation analyses to investigate whether regional differences in FA could be potentially associated with the variance in clinical symptom ratings in each genotype group. To assess correlation analysis, the DTI data were analyzed using the TBSS General Liner Model (GLM) regression analysis with PANSS scores and DST scores as a factor. To further reduce the possibility of false-positive results, only clusters with more than 100 contiguous voxels were considered in the analysis.

### Statistical Analysis of Other Variables

To compare demographic data between schizophrenia and HC subjects, an independent t-test and Pearson Chi-square test were used. In addition, for schizophrenia patients, an independent t-test, Pearson Chi-square test, and Fisher’s exact test were performed to compare demographic and clinical data between the two genotype groups (rs2312147 CC vs. CT/TT). Statistical analysis was performed using IBM SPSS Statistics version 21 (IBM SPSS Statistics, Somers, New York) and p<0.05 was considered statistically significant.

## Results

### Characteristics of study subjects

Table 1 summarizes the characteristics of the study subjects. There was no significant difference in characteristics, including age, sex, and brain volumes, between the schizophrenia and HC groups, except for years of education.

**Table 1:** Characteristics of study subjects.

	Schizophrenia	HC	t	df	p
	(n = 36)	(n = 18)			
Sex, male/female (n)	11/25	8/10			0.31^a^
Age at scan (years, mean±SD)	29.61±9.75	32.00±6.77	−0.93	52	0.36
Education (years, mean±SD)	12.67±2.94	15.08±3.04	−2.82	52	0.01
Brain Volumes (ml, mean±SD)					
ICV	1462.10±160.49	1542.46±223.84	−1.52	52	0.14
GM/ICV	43.99±3.29	42.69±3.26	1.37	52	0.18
WM/ICV	37.70±3.15	38.64±3.92	−0.95	52	0.35
CSF/ICV	18.31±4.01	18.68±4.75	−0.30	52	0.77

CSF, Cerebrospinal Fluid; GM, gray matter; ICV, intracranial volume; SD, standard deviation; WM, white matter.

a Pearson Chi-square test.

### Genotyping

Genotype distributions of all 54 subjects were in accordance with Hardy-Weinberg equilibrium (χ^2^ = 0.01, df = 1, p = 0.91). No statistically significant association between schizophrenia and the rs2312147 genotype was observed among participants in this study (p = 0.89).

Genotype distributions of all 36 patients with schizophrenia (CC: n = 18, CT: n = 15, TT: n = 3) were in accordance with Hardy-Weinberg equilibrium (χ^2^ = 0.003, df = 1, p = 0.96). When we divided the patients with schizophrenia into the CC and CT/TT genotype groups, subjects in the respective genotype groups were not significantly different in terms of sex (p = 0.47) and age (p = 0.79). Scores on all rating scales were not significantly different according to rs2312147 genotype (Table 2).

**Table 2:** Comparison of the sociodemographic and clinical characteristics between rs2312147 genotype groups in patients with schizophrenia.

	CC	CT/TT	t	df	p
	(n = 18)	(n = 18)			
Sex, male/female (n)	7/11	4/14			0.47^a^
Age at scan (years, mean±SD)	29.17±10.50	30.06±9.22	−0.27	34	0.79
Education (years, mean±SD)	12.61±2.64	12.72±3.29	−0.11	34	0.91
Duration of Illness (years, mean±SD)	3.11±4.91	1.81±2.88	0.97	34	0.34
Medication dose at scan (mg, mean±SD)^b^	777.8±215.7	811.1±232.4	−0.45	34	0.66
Medication duration at scan (days, mean±SD)	9.06±8.25	5.94±5.90	1.28	33	0.21
Kinds of antipsychotics at scan (n)					0.32^c^
Paliperidone	17	13			
Olanzapine	0	1			
Risperidone	0	1			
Drug-Naive	1	3			
Brain Volumes (ml, mean±SD)					
ICV	1447.49±123.10	1476.72±193.45	−0.54	28.8	0.59
GM/ICV	44.43±3.78	43.54±2.75	0.81	34	0.43
WM/ICV	37.89±2.13	37.52±3.98	0.34	34	0.73
CSF/ICV	17.68±3.54	18.94±4.44	−0.94	34	0.36
PANSS total score (mean±SD)	107.39±25.89	115.00±19.19	−1.00	34	0.32
DST score (mean±SD)	9.79±3.58	8.60±2.85	0.99	27	0.33
IQ (mean±SD)	101.71±17.70	98.00±20.98	0.51	27	0.61

CC/CT/TT, The genotypes of rs2312147; CSF, Cerebrospinal Fluid; DST, Digit Symbol Test; GM, gray matter; ICV, intracranial volume; IQ, intelligence quotient; PANSS, Positive and Negative Syndrome Scale; SD, standard deviation.

a Fisher’s exact test.

b Antipsychotic medication dose of all patients equivalent to chlorpromazine at the time of scan.

c Pearson Chi-square test.

### VBM results of GM

According to the VBM analysis of GM volumes, there was no significant difference between patients with schizophrenia and HC subjects. In addition, no differences between CC and CT/TT genotype groups were found in patients with schizophrenia. Two-way ANOVA with age, sex, and ICV as covariates showed no significant diagnosis-by-genotype interaction between schizophrenia and HC subjects. No significant voxelwise correlation was found between GM volumes and clinical data in each genotype group.

### TBSS results of WM

**Figure 1:**
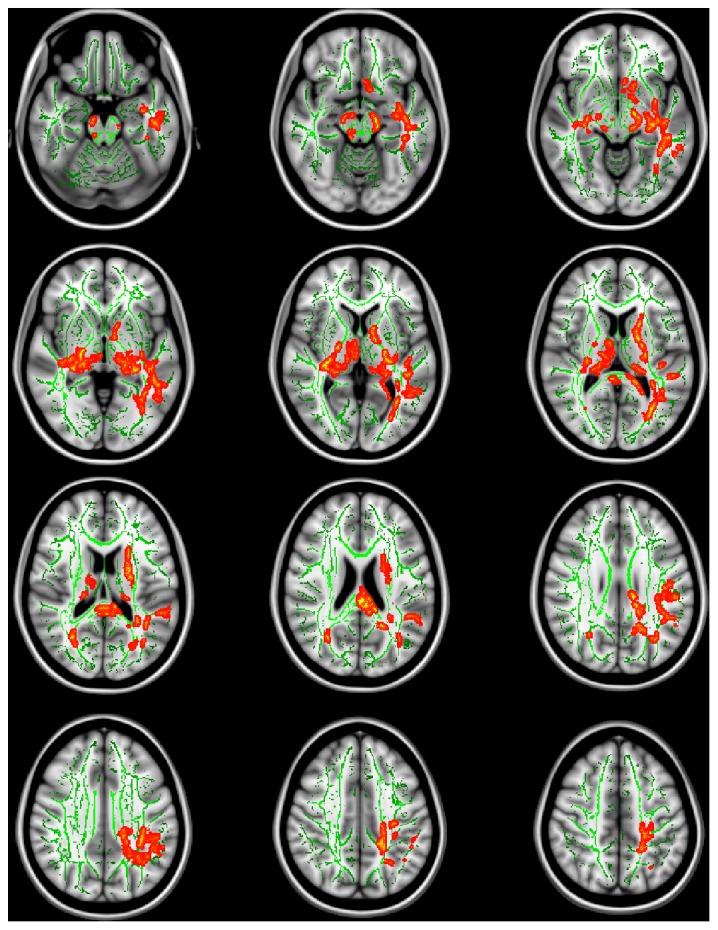
Tract-Based Spatial Statistics (TBSS) analysis showing significant differences in fractional anisotropy (FA) values between the rs2312147 CC genotype group and the CT/TT genotype group in patients with schizophrenia. Voxels demonstrating significant increases in FA values for the CC genotype group compared with the CT/TT genotype group are shown in red-yellow (TFCE-corrected p<0.05). Results are overlaid on the Montreal Neurologic Institute 1 mm template (Z = −20 to Z = 46) and the mean FA skeleton (green). A threshold-free cluster enhancement method was applied using a permutation-based inference tool for nonparametric statistics. The number of permutations was 10,000. Left-right orientation is according to radiological convention.

There was no significant difference between schizophrenia and HC with regard to FA value. Results of TBSS analysis between the CC and CT/TT genotype groups in schizophrenia patients yielded four clusters of significant (TFCE-corrected p<0.05) voxels on the WM skeleton. However, no regions of significantly different FA values between the two genotype groups were found within HC subjects, and there was no significant diagnosis-by-genotype interaction according to two-way ANOVA with age and sex as covariates. According to the group comparison of FA between the CC and CT/TT genotype groups in schizophrenia patients, the FA value was significantly higher (TFCE-corrected p<0.05) in the rs2312147 CC genotype group than in the CT/TT genotype group in four clusters of significant voxels on the WM skeleton (Figure 1). For each cluster, the total number of voxels, the Z-value, peak coordinates, and the anatomic locations are listed in Table 3. The largest cluster (6967 voxels) was located in the splenium of corpus callosum, the left occipital lobe WM, the left anterior limb of the internal capsule, the left temporal lobe WM, and the left fornix/stria terminalis. The second cluster (1565 voxels) was located in the right retrolenticular part of the internal capsule. And the third (181 voxels) and the fourth (163 voxels) clusters were located in the left cingulate gyrus, the left parietal lobe WM, and the right temporal lobe WM. Including age and sex as covariates in the analysis did not influence the obtained results.

**Table 3:** Regions showing significant increases of fractional anisotropy (FA) value in rs2312147 CC genotype group compared to CT/TT genotype group in patients with schizophrenia.

Cluster size (voxels)	Peak coordinates (mm)^a^	Z	Anatomical locations	p^b^
6967	−1, −31, 22	4.6	Splenium of corpus callosum	0.039
	−26, −75, 13	4.6	Occipital lobe white matter, left	0.039
	−21, 13, 12	4.5	Anterior limb of internal capsule, left	0.043
	−56, −54, 21	4.2	Temporal lobe white matter, left	0.047
	−29, −30, −3	4.2	Fornix (cres)/Stria terminalis, left	0.040
1565	29, −23, 3	4.2	Retrolenticular part of internal capsule, right	0.039
181	−18, −54, 29	3.3	White matter in the cingulate gyrus, left (adjacentto the posterior corona radiata, left)	0.049
	−17, −54, 24	2.5	Parietal lobe white matter, left (adjacent tothe splenium of corpus callosum)	0.049
163	31, −61, 23	5.3	Temporal lobe white matter, right	0.043
	30, −64, 19	4.7	Temporal lobe white matter, right (includingthe posterior corona radiata, right)	0.043
	29, −66, 17	3.4	Temporal lobe white matter, right (includingthe posterior thalamic radiation, right)	0.044

Note: Permutation-based inference tool for nonparametric statistics, threshold-free cluster enhancement (TFCE) method was used. Number of permutation was 10,000. To further reduce the possibility of false-positive results, only clusters with more than 100 contiguous voxels were considered in the analysis.

CC/CT/TT, The genotypes of rs2312147.

a Foci for significant differences are listed (TFCE-corrected p<0.05).

b TFCE-corrected p value.

We analyzed voxelwise correlation analyses between clinical data and the FA values of the WM clusters that showed significant group differences between the CC and CT/TT genotypes in schizophrenia. There was no significant correlation between the FA values and PANSS scores in each genotype group of schizophrenia. To investigate the relation between the cognitive function and the WM connectivity with regard to the rs2312147 genotype group in schizophrenia, correlation analyses were conducted in each group. DST scores (age corrected) were shown to be positively correlated with the FA value among patients in the rs2312147 CT/TT genotype group. The related brain regions are listed in Table 4. There was no significant correlation between FA values and DST scores in the CC genotype group.

**Table 4:** Regions showing significant positive correlation between Digit Symbol Test (DST) scores (age corrected) and fractional anisotropy (FA) in the rs2312147 CT/TT group of schizophrenia, in identified regions where significant group differences between the CC and CT/TT genotypes in schizophrenia were found.

Cluster size (voxels)	Peak coordinates (mm)^a^	Anatomical locations	p^b^
958	−37, −20, −6	Sagittal stratum, left	0.028
	−33, −16, −11	Fornix (cres)/Stria terminalis, left	0.030
333	−17, −2, 9	Posterior limb of internal capsule, left	0.033
	−18, 0, 10	Anterior limb of internal capsule, left	0.033
199	−49, −38, 12	Temporal lobe white matter, left	0.040
177	−20, −39, 32	Frontal lobe white matter, left (includingposterior corona radiata, left)	0.037
	−18, −38, 30	Cingulate gyrus, left (including the spleniumof corpus callosum)	0.038
138	−13, −26, 28	Cingulate gyrus, left (including the body ofcorpus callosum)	0.039
138	−47, −21, −18	Temporal lobe white matter, left	0.044

Note: Permutation-based inference tool for nonparametric statistics, threshold-free cluster enhancement (TFCE) method was used. Number of permutation was 10 000. To further reduce the possibility of false-positive results, only clusters with more than 100 contiguous voxels were considered in the analysis.

CC/CT/TT, The genotypes of rs2312147.

a Foci for significant differences are listed (TFCE-corrected p<0.05).

b TFCE-corrected p value.

## Discussion

Recently conducted GWASs showed the significant association of rs2312147, located about 50 kb upstream of VRK2, and schizophrenia in Asians [9] as well as Europeans [2], [5], [10]. Here, we first report a significant association of rs2312147 with the WM connectivity in schizophrenia patients. Furthermore, the FA values of the CT/TT genotype group of rs2312147 were positively correlated with DST scores (age corrected), which are reported to reflect generalized dysfunction that may cause the widespread cognitive failures in schizophrenia [24], [25].

It has been suggested that VRK2 might play important roles in brain development [9]. In a similar context, we demonstrated that the WM connectivity of several brain regions could differ with regard to rs2312147 genotype groups in this study. Between rs2312147 CC and CT/TT genotype groups, the significantly different WM connectivity was found in the splenium of corpus callosum, the left occipital lobe WM, the internal capsule (left anterior limb and right retrolenticular part), the bilateral temporal lobe WM, the left fornix/stria terminalis, the left cingulate gyrus WM, and the left parietal lobe WM. The current findings suggest that extensive WM regions could be associated with the rs2312147 genotype in patients with schizophrenia. Since underlying mechanisms may be hard to know until now, this needs to be tested in future studies.

Although the exact mechanism should be unveiled in further studies, the association of VRK2 with schizophrenia has been replicated in GWASs and a meta-analysis [2], [5], [9], [10]. Moreover, Li et al. [9] suggested that the decreases in WM volume, as well as total brain volume, were significantly associated with rs2312147 (VRK2) in healthy subjects. Meanwhile, another GWAS [14] revealed the association between the SNPs around VRK2 (rs13026414 and rs2717068) and genetic epilepsies, which may suggest VRK2-associated neurodevelopmental alterations. In addition, the impairment of cortical development in the 2p15-p16.1 microdeletion syndrome [11], [12], [13] further supports the association between the VRK2 gene, located in the chromosomal region 2p16.1, and neurodevelopmental abnormalities.

The DST scores (age corrected) were related to the FA values of the WM regions showing significant group differences between the rs2312147 CT/TT and CC genotype groups in schizophrenia, including the left sagittal stratum, the left fornix, the left anterior and posterior internal capsule, the left temporal lobe WM, the left frontal lobe WM, and the left cingulate gyrus WM. According to a functional MRI study, performance of DST could be related to activation of the fronto-parietal cortical network [39], which partially overlaps our findings. Furthermore, DST scores have been proposed to have a strong association with WM hyperintensities in patients with dementia [40] and the cognitive impairment observed in schizophrenia [22], [23]. Turken et al.[26] showed a positive correlation between DST scores and the structural integrity of WM tracts, and proposed that DST performance is likely to depend on the properties of the specific WM fiber tracts responsible for signal transduction. Our data support the hypothesis that VRK2 rs2312147 may affect WM connectivity responsible for transmitting information, in relation to cognitive function assessed by DST performance in patients with schizophrenia.

Unlike previous studies reporting a significant difference in WM integrity in patients with schizophrenia [41], [42], there were no significant differences in FA values between patients with schizophrenia and HC subjects in our study. This might be related to the fact that schizophrenia patients in our study had a relatively recent onset [duration of illness, 2.47±4.02 (mean ± SD) years]. Several studies have shown no differences between patients with recent-onset schizophrenia and HC [43], [44]. Friedman et al. [44] suggested that there might be less widespread changes in WM at the onset of schizophrenia which progresses to more chronic states. To find possible relationships between FA values and the duration of illness in our sample, we used TBSS to conduct a voxelwise correlation analysis between FA values and the duration of illness in patients with schizophrenia. There was a trend towards negative correlation between FA values and duration of illness in patients with schizophrenia (uncorrected p = 0.03; TFCE-corrected p = 0.32) in accordance with a previous study [41], which might be a potential reason for the failure to find differences between the patients with schizophrenia and the HC subjects.

An association between FA values and VRK2 rs2312147 genotypes was found only in the schizophrenia group. VRK2 has been suggested to be involved in signal transduction [16], preventing apoptosis [45], and maintaining nuclear architecture [46]. The serine/threonine kinase binds JIP1 [Jun NH2-terminal Kinase (JNK) Interacting Protein 1] [16], which in neuronal cells plays a role as an anti-apoptotic factor during stress [47] and contributes to axonal development [48]. Altered apoptosis has been proposed as a potential mechanism involved in abnormal neurodevelopment and neurodegenerative processes associated with schizophrenia [49]. Considering the possible role of VRK2 in apoptosis, it can be assumed that the WM connectivity abnormalities associated with VRK2 rs2312147 genotypes may be shown only in patients with schizophrenia. However, this should be interpreted with caution since the effect of VRK2 on the brain structure of patients with schizophrenia has not been investigated until now.

DTI is widely used to characterize WM micro-architecture and brain connectivity in vivo. However, DTI suffers serious limitations in regions with crossing fibers since traditional tensor techniques cannot represent multiple, independent intra-voxel orientations [50]. Several complementary algorithms have been proposed to resolve crossing fibers [50], [51], [52]. However, as these approaches are highly sensitive to noise, authors typically suggest limiting their application to areas of known fiber crossing to avoid erroneous detections [52]. Above all, many of the algorithms require hardware constraints, lengthy acquisition sequences, or long computation times [51] that limit widespread adaptation of these methods in clinical research. Since complementary methods to resolve crossing fibers could not be used in our analysis as a result of the above-mentioned limitations of those methods, the possibility remains that crossing fibers may have affected the alterations in FA values in current results.

There are several limitations in our study. First, the sample size in each group was small. Although the primary outcome of the differences in FA between the two genotypes was positive in the patients with schizophrenia, additional analyses comparing FA values between SZ and HC groups showed negative results. However, our failure to find significant differences according to the diagnosis may be a reflection of a small sample size, and resultant limitations in statistical power. The power of our sample to detect differences in FA between SZ and HC groups was calculated using a two-tailed alpha value of 0.05 and 80% power. With these parameters and considering the genotype frequencies in our sample, the power analysis showed that our sample size had a power (80%) to detect only large effects (d = 0.82), which means that the current findings comparing FA values between SZ and HC groups were inconclusive. Further studies should include a larger number of patients for more accurate results. Second, there has been no association study between schizophrenia and VRK2 rs2312147 in Korean samples yet. Even though prior association results of rs2312147 with schizophrenia were found as significant in samples of Han Chinese [9], the present results in a Korean sample should be interpreted with caution. Third, the influence of medication on brain WM connectivity across genotype groups could not be corrected completely. Although patients had only been on medication for a few days at scan, undetected factors other than the genotype itself might contribute to the observed WM changes in VRK2 rs2312147 genotype group. Fourth, more female subjects were included in the schizophrenia group than in the HC group. Although the sex distribution was not statistically different between the two groups, and including age and sex as covariates in the ANCOVA analysis did not influence the obtained results, a slightly biased sex distribution might be a limitation of this study.

In conclusion, the present study is the first to demonstrate an association between altered WM connectivity and rs2312147 (VRK2) in patients with schizophrenia. Our findings suggest that VRK2 rs2312147 may play a crucial role in the pathophysiology of schizophrenia in association with cognitive function. To unveil the relationship between the VRK2 gene and neurodevelopment in schizophrenia, including its exact mechanism, further replication studies with larger samples will be needed.

## Supporting Information

Figure S1 **Flow diagram of participation of subjects in this study.** CC/CT/TT, The genotypes of rs2312147; DSM-IV-TR, Diagnostic and Statistical Manual of Mental Disorders, 4th Edition, Text Revision; DST, Digit Symbol Test; HC, healthy control; MRI, magnetic resonance imaging; PANSS, Positive and Negative Syndrome Scale; TBSS, Tract-Based Spatial Statistics; VBM, Voxel Based Morphometry; VRK2, vaccinia-related kinase 2 gene.
(PPT)
