# Supplementary material for: Effects of VRK2 (rs2312147) on White Matter Connectivity in Patients with Schizophrenia
Source: PLoS One. 2014 Jul 31;9(7):e103519. doi: 10.1371/journal.pone.0103519 (PMC4117506; doi:10.1371/journal.pone.0103519)
Supplement: Figure S1 — Flow diagram of participation of subjects in this study. CC/CT/TT, The genotypes of rs2312147; DSM-IV-TR, Diagnostic and Statistical Manual of Mental Disorders, 4th Edition, Text Revision; DST, Digit Symbol Test; HC, healthy control; MRI, magnetic resonance imaging; PANSS, Positive and Negative Syndrome Scale; TBSS, Tract-Based Spatial Statistics; VBM, Voxel Based Morphometry; VRK2, vaccinia-related kinase 2 gene. (PPT) [file pone.0103519.s001.ppt]

## Slide 1
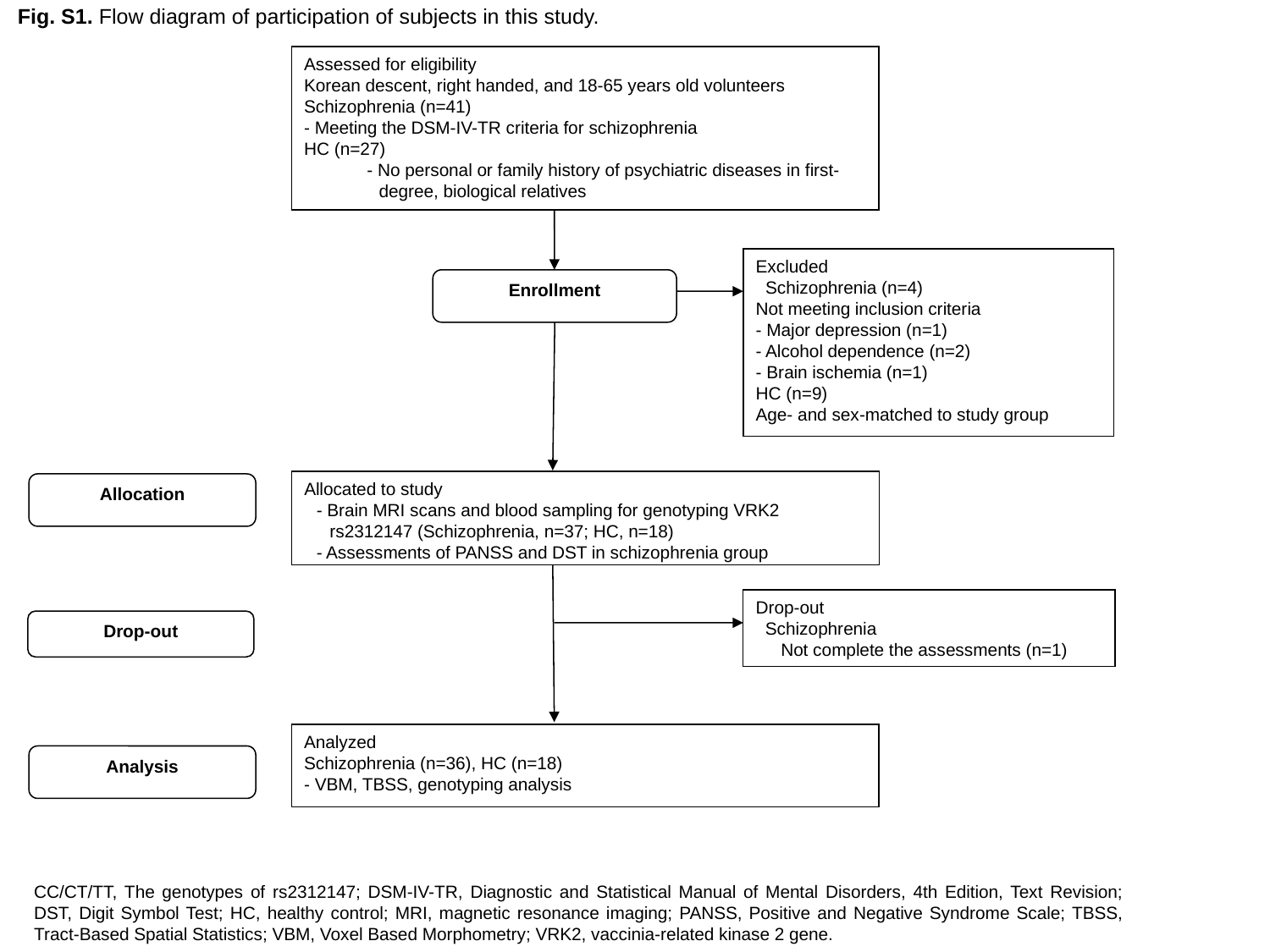

Fig. S1. Flow diagram of participation of subjects in this study.
Assessed for eligibility
Korean descent, right handed, and 18-65 years old volunteers
Schizophrenia (n=41)
- Meeting the DSM-IV-TR criteria for schizophrenia
HC (n=27)
- No personal or family history of psychiatric diseases in first-degree, biological relatives
Excluded
 Schizophrenia (n=4)
Not meeting inclusion criteria
- Major depression (n=1)
- Alcohol dependence (n=2)
- Brain ischemia (n=1)
HC (n=9)
Age- and sex-matched to study group
Enrollment
Allocated to study
- Brain MRI scans and blood sampling for genotyping VRK2 rs2312147 (Schizophrenia, n=37; HC, n=18)
- Assessments of PANSS and DST in schizophrenia group
Allocation
Drop-out
 Schizophrenia
Not complete the assessments (n=1)
Drop-out
Analyzed
Schizophrenia (n=36), HC (n=18)
- VBM, TBSS, genotyping analysis
Analysis
CC/CT/TT, The genotypes of rs2312147; DSM-IV-TR, Diagnostic and Statistical Manual of Mental Disorders, 4th Edition, Text Revision; DST, Digit Symbol Test; HC, healthy control; MRI, magnetic resonance imaging; PANSS, Positive and Negative Syndrome Scale; TBSS, Tract-Based Spatial Statistics; VBM, Voxel Based Morphometry; VRK2, vaccinia-related kinase 2 gene.
